# Bayesian Inference-Based Gaussian Mixture Models With Optimal Components Estimation Towards Large-Scale Synthetic Data Generation for *In Silico* Clinical Trials

**DOI:** 10.1109/OJEMB.2022.3181796

**Published:** 2022-06-10

**Authors:** Vasileios C. Pezoulas, Nikolaos S. Tachos, George Gkois, Iacopo Olivotto, Fausto Barlocco, Dimitrios I. Fotiadis

**Affiliations:** Unit of Medical Technology and Intelligent Information Systems, Department of Materials Science and EngineeringUniversity of Ioannina 37796 GR45110 Ioannina Greece; Department of Experimental and Clinical MedicineUniversity of Florence 9300 50121 Florence Italy; Unit of Medical Technology and Intelligent Information Systems, Department of Materials Science and EngineeringUniversity of Ioannina 37796 GR45110 Ioannina Greece; Department of Biomedical ResearchFORTH-IMBB 69153 GR45110 Ioannina Greece

**Keywords:** Gaussian Mixture Models, synthetic data generator, $in silico$ clinical trials, computational complexity, hypertrophic cardiomyopathy

## Abstract

*Goal*: To develop a computationally efficient and unbiased synthetic data generator for large-scale *in silico* clinical trials (CTs). *Methods:* We propose the BGMM-OCE, an extension of the conventional BGMM (Bayesian Gaussian Mixture Models) algorithm to provide unbiased estimations regarding the optimal number of Gaussian components and yield high-quality, large-scale synthetic data at reduced computational complexity. Spectral clustering with efficient eigenvalue decomposition is applied to estimate the hyperparameters of the generator. A case study is conducted to compare the performance of BGMM-OCE against four straightforward synthetic data generators for *in silico* CTs in hypertrophic cardiomyopathy (HCM). *Results:* The BGMM-OCE generated 30000 virtual patient profiles having the lowest coefficient-of-variation (0.046), inter- and intra-correlation differences (0.017, and 0.016, respectively) with the real ones in reduced execution time. *Conclusions:* BGMM-OCE overcomes the lack of population size in HCM which obscures the development of targeted therapies and robust risk stratification models.

## I. Introduction

Virtual population/synthetic data generation has gained attention in the healthcare sector due to the overwhelming need to overcome the significant lack of sufficient population size, particularly for *in silico* clinical trials, where the orchestration of viable Phase II/III clinical trials (CTs) by pharmaceutical companies is leveraged by the need for expensive drugs [1]–[3]. Furthermore, the lack of medical databases with increased statistical power (e.g., in rare diseases) obscures the deployment of machine learning pipelines which can identify risk factors for disease progression and treatment due to the reduced amount of available training data. As a matter of fact, all these factors have a significant negative impact in the capacity of the healthcare systems, where the costs and delays for treatment and re-admission are already high. Virtual population generation envisages to address these needs through the development of synthetic data generators which are trained on the real datasets to produce virtual (or synthetic) distributions which can “mimic” the real ones in terms of reduced divergence and dispersion with the real data. Since the synthetic data quality is affected by the quality of the real data, it is first necessary to enhance the raw data quality in terms of data completeness and conformity.

Several studies have been launched towards the design of efficient synthetic medical data generators based on both probabilistic approaches, such as, the multivariate normal distribution (MVND) and the Bayesian networks (BN), as well as, machine learning approaches, such as, the artificial neural networks (ANNs), the supervised tree ensembles (STE), and the unsupervised tree ensembles (UTE). The MVND was applied in [4], [5] to generate virtual data based on the mean and the covariance of the real data. In addition, the BN were used in [6]–[8] for the generation of synthetic distributions based on the modeling of conditional probabilities across diverse network topologies. The BN and the MVND, however, suffer from mathematical assumptions; the MVND algorithm assumes that the real data are normally distributed whereas in the BNs the conditional probabilities are modeled using assumptions on the prior distribution of the features. To this end, machine learning based generators have been proposed [9]–[11], such as, the ANNs with radial basis functions [9], [11], the STE [10], [11], and the UTE [10], [11], yielding favorable performance against the probabilistic approaches. However, they are not computationally efficient since they require increased training/testing time. Moreover, the STE, and the ANN [9]–[11] are supervised learning algorithms that require a “target feature” (i.e., an outcome) which influences the correlation of the synthetic data and thus introduces critical biases. Moreover, in the BN, there is an infinite number of edge permutations in one topology which must be pre-defined prior to the simulation.

The design of computationally efficient and unbiased synthetic data generators is a technical challenge, particularly in the case of large-scale CTs. A computationally efficient probabilistic synthetic data generator has been introduced in [12], [13], where Gaussian Mixture Models (GMMs) were used to generate synthetic data. Since GMM maximizes only the data likelihood based on the expectation maximization (EM) approach, it might yield specific structures that might not apply to the data. This can be solved by variational inference (VI) [14]–[16] which is more efficient than EM and reduces the computational complexity. Other attempts [17], [18] focused on the automated adjustment of Gaussian distributions for background modeling. However, none of these studies has focused on the optimal selection of the number of Gaussian components which is arbitrary and affects the estimation of the weight concentration (or gamma) parameter which is of great importance since it affects the log-likelihood of the model.

In this work, we focus on the optimal estimation of the Gaussian components in the BGMM algorithm to yield concrete estimations of the VI at reduced computational complexity for large-scale synthetic data generation (we refer to this approach as BGMM with Optimal Components Estimation: BGMM-OCE). To do so, we first apply spectral clustering based on the Locally Optimal Block Preconditioned Conjugate Gradient (LOBPCG) method to identify the best clustering solution as the one with the highest Davies Bouldin score (DBS) at small complexity. Then, we set the optimal number of clusters as the number of Gaussian components, and we define an exponentially decaying gamma value. The BGMM-OCE's performance was compared against state-of-the-art synthetic data generators (BN, UTE, STE, ANNs) in the context of *in silico* clinical trials for HCM. According to our results, the BGMM-OCE was able to generate 30000 virtual patients having the lowest coefficient of variation (0.046) and goodness of fit (0.191) at small execution time.

## II. Materials and Methods

### A. Outline

**Fig. 1.**
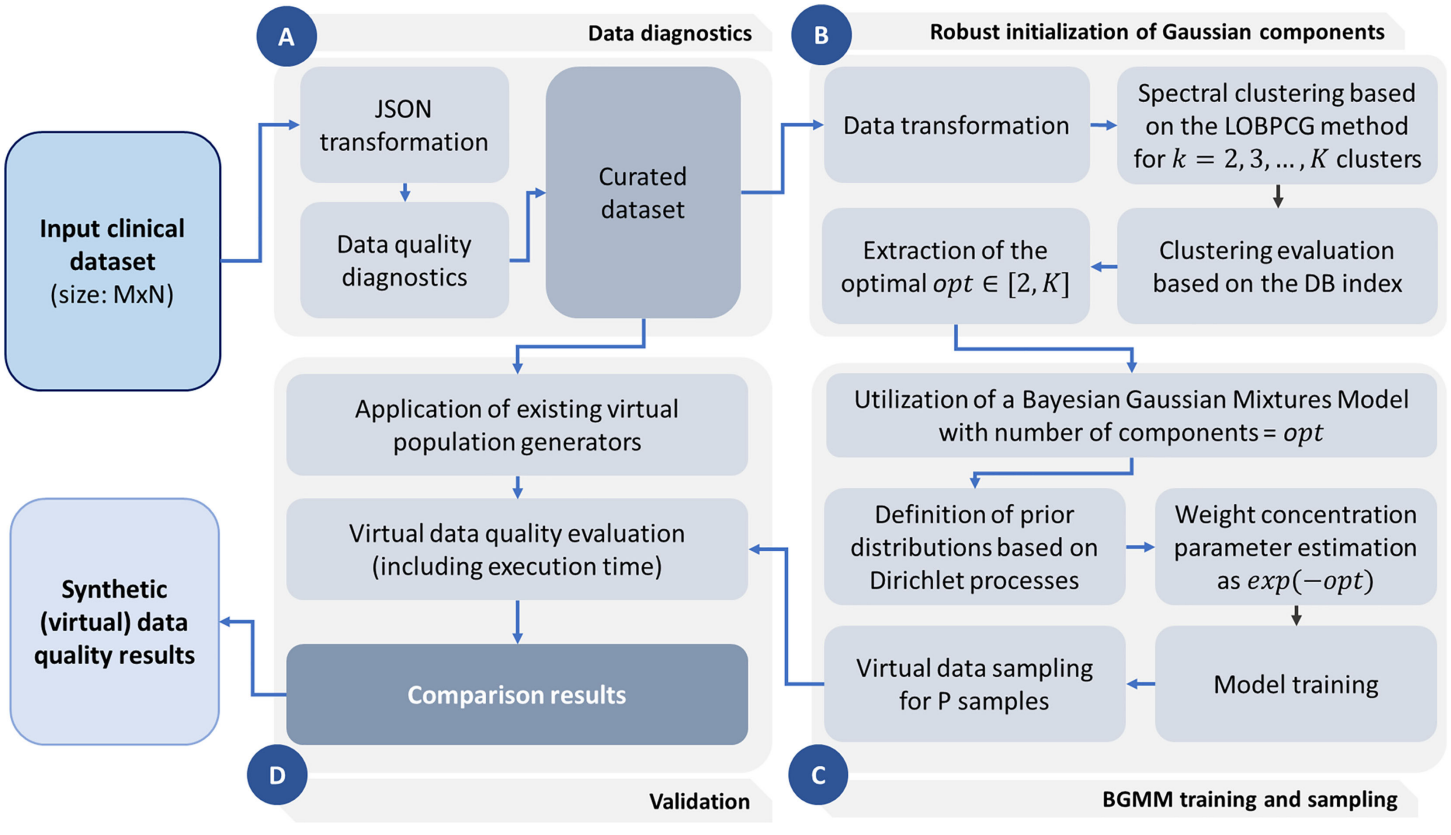
An outline of the BGMM-OCE workflow.

According to Fig. 1, the large-scale synthetic data generation process consists of four stages, namely: (i) the data diagnostics stage, (ii) the robust initialization of Gaussian components stage, (iii) the BGMM training and sampling stage, and (iv) the validation stage. In the first stage, the data are transformed into a JSON format for faster processing. A data diagnostics pipeline is then applied to remove anomalies and address missing values along with further incompatibilities within the raw data. Spectral clustering is then applied on the transformed dataset based on the LOBPCG method to derive $k$-clusters, where $k \in [ {2,K} ]$. The optimal number of clusters is extracted as the one with the highest Davies Bouldin score (DBS), say $opt$. The BGMM is then trained on the transformed (and curated) data, where the number of components is set to $opt$, the prior distribution is based on Dirichlet processes, and the weight concentration (gamma) parameter is set to $exp( { - opt} )$.

Upon the finalization of the BGMM training process, the estimated component hyperparameters are used to randomly sample $P$ multidimensional data samples which refer to the synthetic dataset. The latter is compared with those obtained by straightforward synthetic data generators (validation stage), such as, the BN, STE, UTE, RBF-based ANNs (which were described in Section I), in terms of reduced inter- and intra- correlation differences, coefficient of variation (cV) difference, goodness of fit (GOF), and KL-divergence with the real data. The output of the workflow includes a virtual dataset along with a virtual data quality report.

### B. Diagnostics

The raw clinical data were first transformed into a JSON format to enable faster I/O operations. The input feature space was split into “eligible” and “non-eligible” feature sub-spaces, where the “eligible” features were those having less than 30% missing values without inconsistent fields and anomalies after experimentations with the percentage of information loss. The Isolation Forests were trained on non-missing records to identify outliers [19]. The covariance matrix was estimated between each pair of input features to remove duplicated features along with the Levenshtein distance to remove lexically identical features [20]. The k-nearest neighbors (kNN) [21] method was used to impute missing values, where applicable, for the “eligible” features only.

### C. Robust Initialization of Gaussian Components

#### 1) Fast Eigenvalue Decomposition

A scaling approach robust to “hidden” outliers was applied to standardize the input data, where the scaling and centering process was applied independently for each feature according to the median and the interquartile range. The eigensolver was based on the Locally Optimal Block Preconditioned Conjugate Gradient Method (LOBPCG) which is ideal for large symmetric positive definite (SPD) generalized eigenproblems [22]–[24], as described in Supplementary Material (Section A).

#### 2) Clustering Evaluation Based on the DB Score (DBS)

The Davies-Bouldin Score (Supplementary Material, Section B) was evaluated on a set of clusters and the cluster with the highest DBS was selected as the optimal [25].

### D. BGMM Training and Sampling

#### 1) GMM

A Gaussian mixture model (GMM) lies on the assumption that the data originate by a mixture of Gaussian densities [26]. In practice, the expectation maximization (EM) method is used to estimate the hyperparameters of the GMM, say ${\boldsymbol{\theta }}$, by maximizing the data likelihood (Supplementary Material, Sections C and D). However, an issue with EM is that the resulting structural topologies fail to capture the data due to the complexity of the problem. This can be addressed by variational inference based on Dirichlet processes [26]–[28].

#### 2) Weight Concentration Parameter (gamma) Estimation

The precise definition of the weight concentration parameter is challenging. In practice, the weight concentration parameter is defined as the inverse of the number of components. However, this approach introduces biases since it assumes a linear relationship between them. To deal with this, we exponentiate the optimal number of components to capture non-linear effects, as $exp( { - opt} )$ and we set it equal to gamma.

#### 3) Model implementation, training, and Random Sampling

A pseudocode of the BGMMOCΕ algorithm is described in Supplementary Material, Section E. The input includes the curated dataset, the number of virtual patients, and the initial parameters of the model. The algorithm first applies spectral clustering process, for a set of $k$ clusters under evaluation, based on the LOBPCG method and extracts the best clustering solution, i.e., the one having the highest DBS, say $opt$. Then, the BGMM training process is initialized, where the number of Gaussian components and the weight concentration parameter are set equal to $opt$, and $exp( { - opt} )$, respectively. Random sampling is applied on the trained model based on Dirichlet distributions to yield the synthetic (virtual) samples.

### E. Validation

Four state-of-the art synthetic data generators [6]–[11] were used for comparison purposes, including the BN, the ANNs, the UTE, and the STE. Five quality indicators (Kullback-Leibler divergence, inter- and intra- correlation difference, goodness of fit - GOF, coefficient of variation - cV) [29]–[31] were used to measure the similarity, dispersity, and divergence between the synthetic and the real data (Supplementary Material, Section F).

## III. Results

### A. Data Origins and Related Diagnostics

Anonymized clinical data were acquired by 648 patients with hypertrophic cardiomyopathy as part of the SILICOFCM project [32], [33] (Supplementary Material, Section G).

### B. Large Scale Virtual Population Generation

#### 1) Estimation of the Number of Gaussian Components

**Fig. 2.**
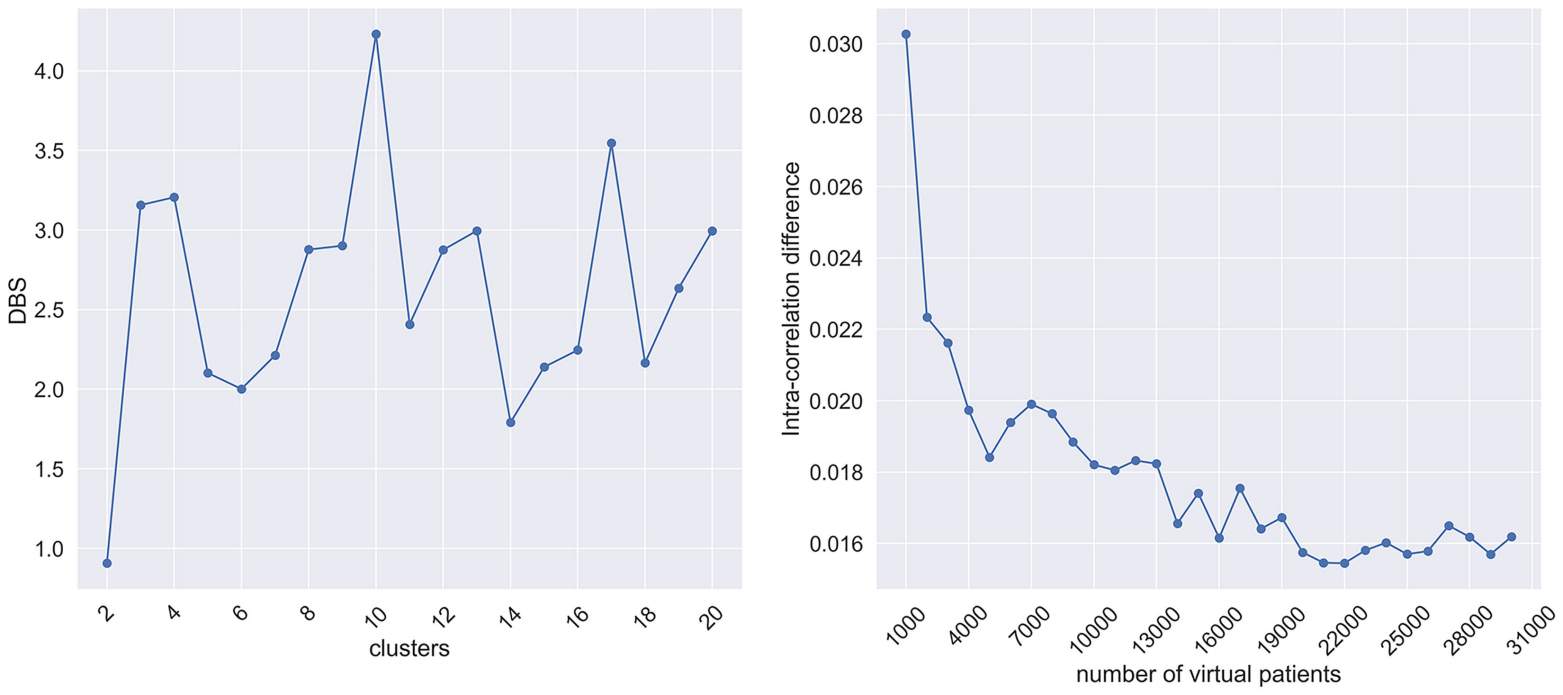
The DBS distribution (left), average intra-correlation difference between the real and the synthetic data for multiple virtual patients (right).

Spectral clustering was first applied to estimate the number of clusters using the LOBPCG eigensolver across a pre-defined number of $k$-clusters, where $k \in [ {2, 20} ]$. The DBS was computed for each cluster to assess the clustering consistency. According to Fig. 2, the number of clusters having the highest DBS was 10. The process was repeated for multiple virtual populations (1000 to 30000 with step 1000). In each case, the BGMM-OCE was trained using 10 Gaussian components. The distribution of the average intra-correlation differences appears to be decaying over the increasing number of virtual patients, with less than 0.018 difference for more than 14000 virtual patients.

#### 2) Comparison With SoA Data Generators

**Fig. 3.**
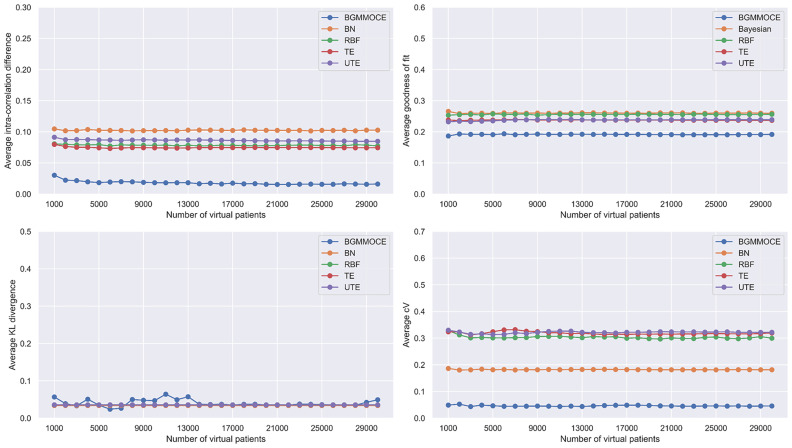
Average intra-correlation (on top left corner), GOF (on top right corner), KL-divergence (on bottom left corner) and cV (on bottom right corner) across multiple virtual patients per data generator.

The virtual data quality results for each data generator are depicted in Fig. 3 across multiple virtual patient scenarios. According to Table I and Fig. 3, the BGMM-OCE achieved the best performance yielding the lowest average intra-and inter-correlation difference, GOF and cV with non-significant variations in the KL-divergence (less than 0.05).

**TABLE I:** Synthetic Data Quality Evaluation Results

**Metric**	**BGMM-OCE**	**BN**	**RBF-based ANNs**	**UTE**	**STE**
**N = 1000**					
**KL-divergence**	0.057	**0.034**	0.036	0.036	0.036
**Goodness of fit**	**0.186**	0.265	0.253	0.232	0.238
**Inter-correlation**	**0.032**	0.110	0.085	0.096	0.084
**Intra-correlation**	**0.030**	0.105	0.081	0.091	0.080
**cV**	**0.049**	0.186	0.328	0.330	0.324
**N = 5000**					
**KL-divergence**	0.035	**0.034**	0.036	0.035	0.035
**Goodness of fit**	**0.186**	0.259	0.257	0.235	0.238
**Inter-correlation**	**0.019**	0.108	0.084	0.091	0.078
**Intra-correlation**	**0.018**	0.102	0.079	0.091	0.074
**cV**	**0.046**	0.181	0.300	0.314	0.324
**N = 10000**					
**KL-divergence**	0.047	**0.034**	0.036	0.035	0.035
**Goodness of fit**	**0.192**	0.259	0.255	0.239	0.238
**Inter-correlation**	**0.019**	0.107	0.082	0.092	0.078
**Intra-correlation**	**0.018**	0.102	0.078	0.087	0.074
**cV**	**0.045**	0.182	0.306	0.325	0.320
**N = 20000**					
**KL-divergence**	0.035	**0.034**	0.036	0.035	0.035
**Goodness of fit**	**0.191**	0.26	0.256	0.238	0.238
**Inter-correlation**	**0.017**	0.108	0.082	0.090	0.078
**Intra-correlation**	**0.016**	0.102	0.078	0.085	0.074
**cV**	**0.046**	0.181	0.297	0.324	0.315
**N = 30000**					
**KL-divergence**	0.049	**0.034**	0.036	0.035	0.035
**Goodness of fit**	**0.191**	0.26	0.256	0.239	0.237
**Inter-correlation**	**0.017**	0.108	0.081	0.089	0.078
**Intra-correlation**	**0.016**	0.103	0.077	0.085	0.074
**cV**	**0.046**	0.181	0.300	0.322	0.319

**Fig. 4.**
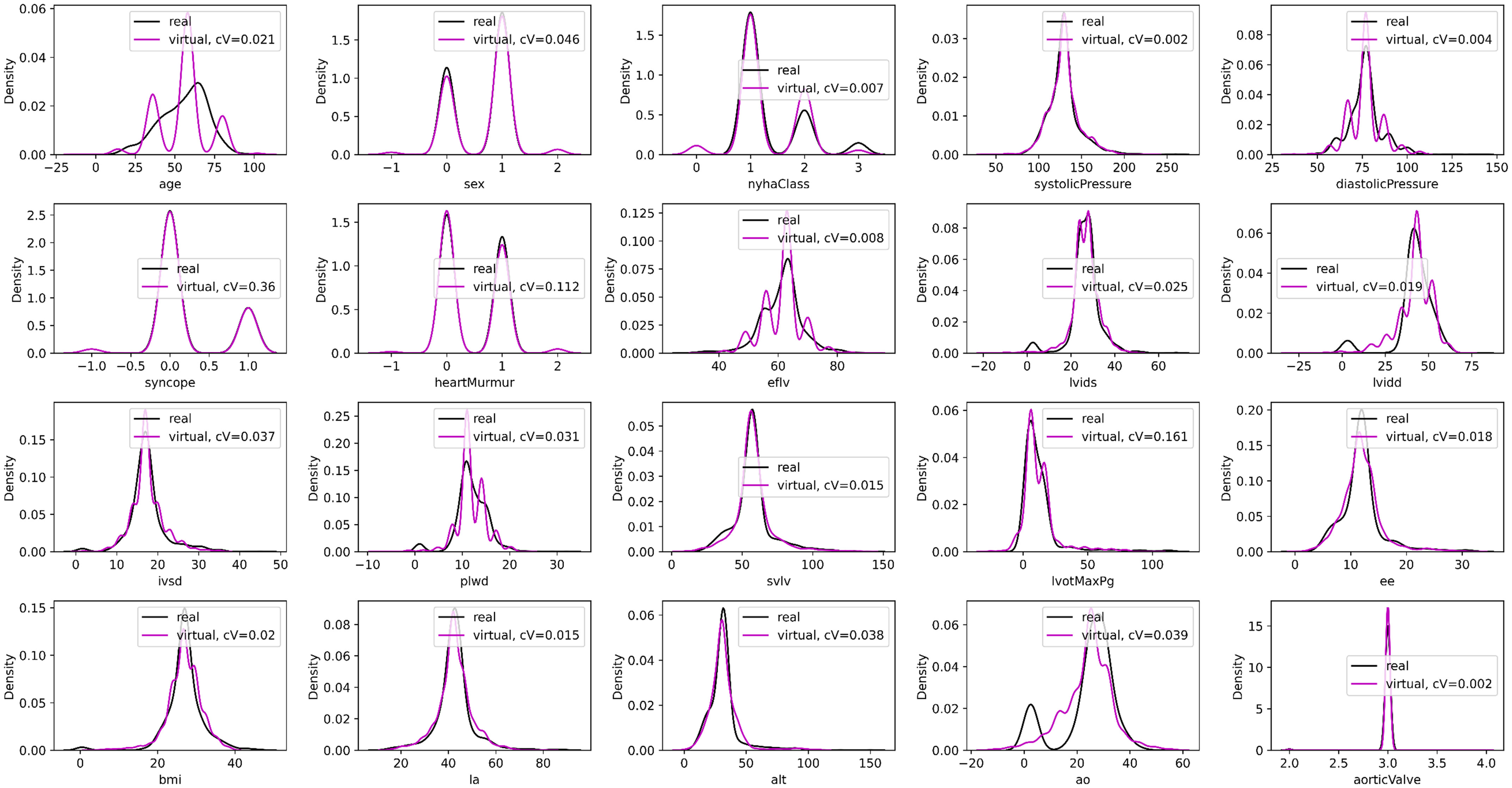
An illustration real (black) and synthetic (magenta) distributions for the 20 features under evaluation (Supplementary Material, Section G) for 1000 virtual patients. The cV values refer to the absolute coefficient of variation difference between the real and the synthetic distributions.

Gaussian kernel density estimation was applied to estimate the density of the real and synthetic data. According to Fig. 4, the synthetic distributions tend to “mimic” the real ones. In all cases, the average cV difference between the real and the synthetic distributions was less than 0.1 (for 1000 virtual patients) highlighting the reduced dispersity of the synthetic data with respect to the real distributions.

To further demonstrate the biases which are introduced in the case where the weight concentration parameter is set equal to $1/opt$, we applied additional BGMM-OCE experimentations. According to Supplementary Table II, both the average correlation difference between the real and the virtual patients (intra-correlation difference) and the average correlation difference between the real and the virtual features (inter-correlation difference), over multiple virtually generated patients, is not well preserved, yielding higher differences compared against those from the BGMM-OCE configuration in Table I. The GOF and KL-divergence scores are similar to the values reported in Table I and thus are not reported in Supplementary Table II.

### C. Execution Time

**Fig. 5.**
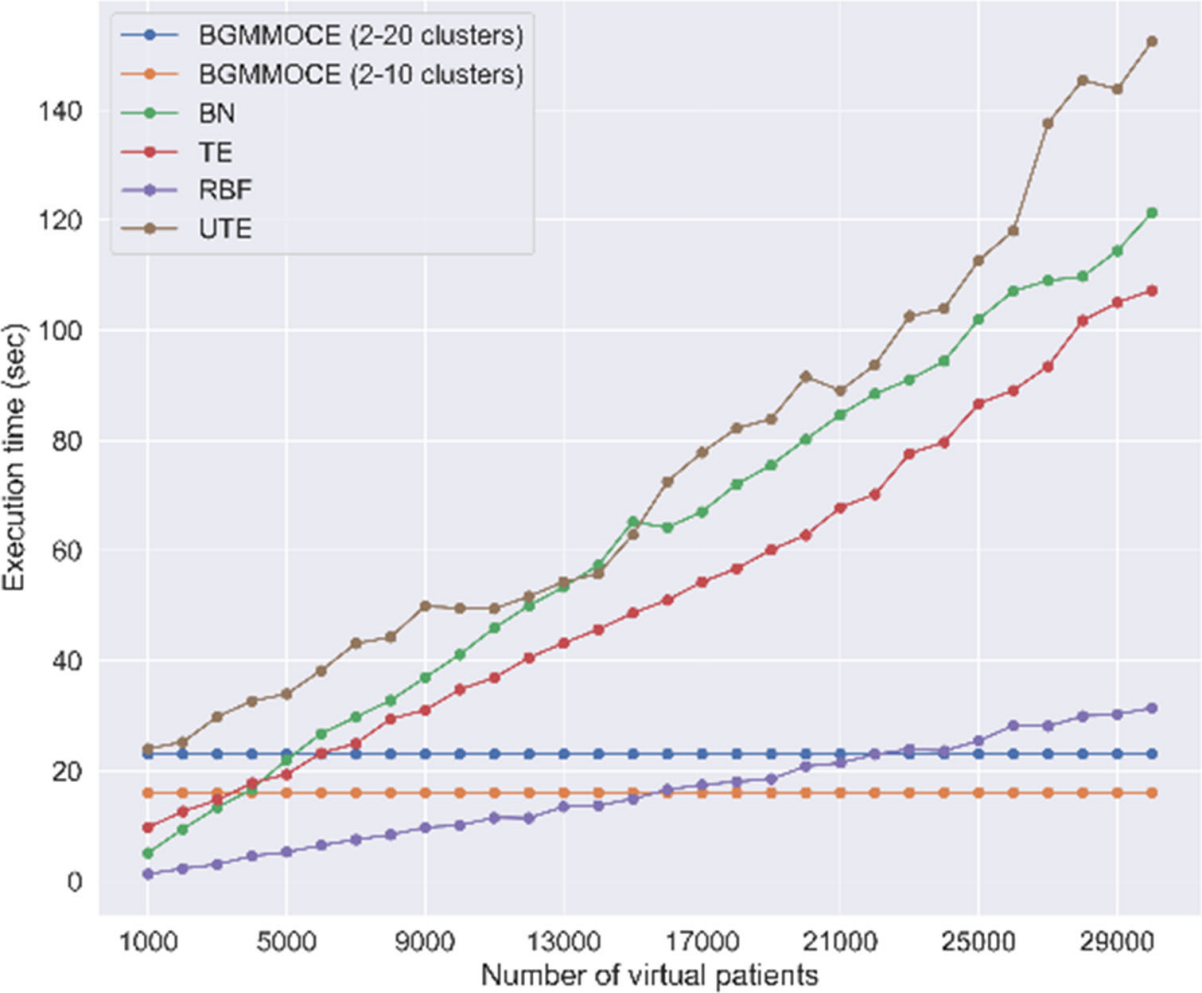
Execution time comparison results.

According to Fig. 5, the BGMM-OCE required 23 secs on average for the optimal component initialization step. In the case where the application of spectral clustering involved 2-10 clusters, the execution time was reduced to 16 sec. However, the execution time for random sampling across different virtual populations was 0.031 sec on average.

The TE, BN, and UTE had the largest average execution time (53 sec, 63 sec, and 75 sec, respectively). Interestingly, the RBF-based ANNs achieved the lowest average execution time (16 sec), but its increased computational tendency for virtual populations beyond 17000 or 23000 patients indicates a higher complexity than BGMM-OCE.

## IV. Discussion

In this work, we developed a robust and computationally efficient large scale synthetic data generator to overcome the lack of sufficient population size and leverage the increased costs for patient recruitment for *in silico* clinical trials. Our intention was to resolve significant biases which are introduced by the estimation of the hyperparameters during the BGMM training process. To do so, we proposed the BGMM-OCE which was designed to: (i) avoid the use of an arbitrary number of Gaussian components through a computationally efficient spectral clustering stage, and (ii) provide non-linear estimation of the gamma parameter by exponentiating the number of components. According to our results, the BGMM-OCE outperformed state-of-the art synthetic data generators, yielding lowest cV, GOF, KL divergence, and inter- and intra- correlation differences at reduced computational complexity.

Spectral clustering is computationally demanding and particularly during the extraction of an increasing number of clusters. To overcome this limitation, we used the LOBPCG method to extract fast estimations of the eigenvectors and eigenvalues by solving the minimum trace problem, rather than using the conventional ARPACK [34] and AMG [35] solvers which are computationally demanding. To further reduce the complexity of the clustering evaluation process, we store the local maxima of the DBS and if there are no reported maxima after 5 clusters under evaluation, the process is terminated thus avoiding additional unnecessary clustering evaluations. The cluster with the highest DBS is then extracted to define the number of Gaussian components in the BGMM training stage. In addition, the gamma parameter was exponentially related (non-linearly) to the number of components, rather than inverse related (linear), to avoid linear assumptions.

Similar to previous studies [11], [16], the BGMM-OCE places particular emphasis on the quality of the input data since lack of data quality reduces the statistical power of the outcomes. Thus, the quality of the real data is reflected on the synthetic data. Here, we extended an automated data curation pipeline presented in [20] to avoid data contamination by separating the features in the input space into two states; the “eligible” and the “non eligible”. Advanced outlier detection methods like the Isolation Forests were used to identify outliers and string-matching methods were applied to detect duplicated features.

According to Supplementary Table I, the MVND and the log-MVND [4], [5], [36] are fast but they are based on critical assumptions (normality) and yield synthetic data with reduced quality. Although the BN offer explainable presentations of the conditional probabilities through the network, the different topologies are infinite, the quality of the virtual data is reduced, and the computational complexity is large [6]–[8]. The STE and UTE yield synthetic data with better quality, but they still have increased computational complexity for training/testing. In addition, they need a “target feature” that influences the correlation of the synthetic features and introduces critical biases. The same stands for the ANN but it has reduced computational complexity. According to Supplementary Table II, the inter- and intra- correlation differences are higher than those reported for the BGMM-OCE in Table I (where the weight concentration parameter was equal to $exp( { - opt} )$). Thus, setting the weight concentration parameter equal to $exp( { - opt} )$ provides more concise and coherent virtual patient profiles with well-preserved correlations among the features.

The UTE, STE, and ANN are unable to capture the inter- and intra- correlation differences. As far as the GMM algorithm is concerned, although it is more computationally efficient, but it requires multiple hyperparameters which are arbitrarily defined [16] and thus they introduce biases. However, the precise definition of components and the estimation of the weight concentration parameter is a technical challenge. The BGMM-OCE overcomes this limitation by introducing a clustering stage based on the LOBPCG method prior to the BGMM training to estimate the optimal number of clusters as the one with the highest DBS across a set of predefined clusters. The best clustering solution is then set equal to the number of Gaussian components, and the weight concentration parameter is exponentially related to the number of Gaussian components instead of assuming linear dependencies. The BGMM-OCE script is available in the following GitHub repository: https://github.com/vpz4/BGMM-OCE along with high-quality synthetic HCM data. We plan in the near future to utilize the BGMM-OCE in additional domains with insufficient population size to make drug testing feasible [36].

## V. Conclusion

BGMM-OCE introduces a highly efficient spectral clustering stage to overcome the definition of arbitrary hyperparameters in the BGMM process. The BGMM-OCE can yield high-quality synthetic data at reduced complexity to enable the design of targeted therapies and the development of disease prediction models.
